# Serial order and complexity of Category Fluency words in an Italian cohort of MCI individuals

**DOI:** 10.1002/alz.088781

**Published:** 2025-01-03

**Authors:** Matteo De Marco, Chiara Da Ronch, Annalena Venneri, Maurizio Gallucci

**Affiliations:** ^1^ Brunel University London, Uxbridge, UB8 3PH United Kingdom; ^2^ Cognitive Impairment Center, AULSS2 Marca Trevigiana, Treviso, 31100 Italy; ^3^ Brunel University London, London United Kingdom; ^4^ University of Parma, Parma Italy

## Abstract

**Background:**

The Category Fluency Test (CFT) is the most widely‐used test of semantic memory (SM). A number of studies indicate that the serial order (SO) in which words are generated during the CFT can be informative of the underlying level of SM integrity. In this study, SO and item‐level complexity of CFT words were investigated for the first time in a cohort of Italian individuals with mild cognitive impairment (MCI).

**Method:**

The CFT (category: animals) was administered to 60 MCI individuals recruited as part of the TREviso DEMentia (TREDEM) registry and to 54 matched healthy controls. Each CFT word was scored according to its graphemic length its typicality within the cohort and, as per Italian normative values, also according to its frequency of use and age of acquisition. Rank‐based correlations were calculated for each individual between SO and each item‐level feature and *z*‐converted for data analysis. One‐way *ANOVA*s were then run to compare these correlational indices between the two groups, controlling for age, education and sex.

**Result:**

A significant between‐group difference emerged from the analysis of the SO‐word length correlational index (*p* < 0.004). Both groups tended to generate increasingly longer words during CFT performance, but in the MCI group this increase was significantly steeper.

**Conclusion:**

While word length does not embed any semantic information, these findings indicate that the graphemic length‐based organisation of lexical‐semantic material during recall is altered in native Italian‐speaking MCI individuals.

